# Dietary patterns and association with Iron deficiency among children and adolescents aged 9–17 years in rural Guangzhou, China: a cross-sectional study

**DOI:** 10.3389/fnut.2024.1443849

**Published:** 2024-09-02

**Authors:** Jinhan Fu, Chunzi Zeng, Jie Huang, Jiaying Guo, Zheng Su, Shiyun Luo, Weiwei Zhang, Zhoubin Zhang, Huilian Zhu, Yan Li

**Affiliations:** ^1^School of Public Health, Sun Yat-sen University, Guangzhou, China; ^2^Department of Foodborne Diseases and Food Safety Risk Surveillance, Guangzhou Center for Disease Control and Prevention, Guangzhou, China; ^3^School of Public Health, Southern Medical University, Guangzhou, China

**Keywords:** children, adolescents, dietary patterns, iron deficiency, Guangzhou, China

## Abstract

**Background:**

Iron deficiency and iron deficiency anemia cause a huge disease burden worldwide. Diet is an important factor affecting the iron levels. This study aims to explore the dietary patterns of school-aged children in rural areas of Guangzhou and their association with iron deficiency.

**Methods:**

Data on dietary surveys, lifestyle, demographic and laboratory tests were gathered from rural school-age children in Guangzhou. Factor analysis was applied to derive dietary patterns. Robust Poisson regression and subgroup analysis were used to analyze the association between dietary patterns and iron deficiency.

**Results:**

A total of 2,530 children and adolescents aged 9–17 years were enrolled. The prevalence of iron deficiency was 13.36%. Four dietary patterns were identified including snack and fast-food pattern, fruit and vegetable pattern, cereal and tuber pattern and meat and offal pattern. Both children and adolescents in the Q4 group (the highest propensity) of snack and fast-food pattern and cereal and tuber pattern had a higher risk of iron deficiency than the Q1 group (the lowest propensity). Both children and adolescents in the Q4 group of meat and offal pattern and fruit and vegetable pattern had a lower risk of iron deficiency than the Q1 group. The results of stratified analysis showed the negative effect of snack and fast-food pattern and the protective benefits of meat and offal pattern are more obvious for boys, and the negative effect of cereal and tuber pattern were obvious for girls. The negative effect or protective benefits of the four dietary patterns were obvious for children aged 9–13.

**Conclusion:**

Females, older children, and those with shorter sleep duration are at higher risk of iron deficiency. Snack and fast-food pattern and cereal and tuber pattern are risk factors for iron deficiency, and fruit and vegetable pattern and meat and offal pattern are protective factors for iron deficiency. The impact of diet on body iron levels is more obvious in boys and younger children. The findings of this study can provide evidence for formulating prevention and control measures on children and adolescents iron deficiency and iron deficiency anemia.

## 1 Introduction

Iron is an essential trace element in the human body, which participates in the transportation of oxygen and tissue respiration, maintains normal hematopoietic function, and maintains immune function. Iron deficiency (ID) is a common micronutrient deficiency (1), which refers to a state of reduced or depleted iron reserves in the body (2). According to the World Health Organization (WHO), 280 million children worldwide suffer from anemia, and ID is one of the main causes of anemia (3). In addition, the United Nations Millennium Development Goals (MDGs) also emphasize the severity of micronutrient deficiency, pointing out that more than 2 billion people lack essential minerals such as iron, which are essential for their growth, development and healthy life (4). From 2000 to 2020, the prevalence of anemia among Chinese children aged 0–14 years was 19.9% (5). A survey conducted in seven cities and two townships in China showed that ID detection rate in child was 35.5% and the anemia rate was 9.2% (6).

ID has a slow onset and is easily overlooked in the early stages. In its incipient stages, ID can impair enzyme activity and disrupt neurotransmitter synthesis and transmission, potentially resulting in symptoms such as mental depression and diminished appetite. As the condition progresses, it can develop into iron deficient anemia (IDA). School-aged Children are more prone to developing ID due to increased nutritional needs during their growth and development stages, leading to anemia, which in turn leads to delayed growth and development, decreased immune function, and brain development damage (7). Therefore, evaluating the iron reserves in school-aged children can move the key to preventing IDA forward, take timely intervention measures, and reduce the harm caused by related diseases.

The iron level in the body is influenced by various factors, including digestive system diseases, physiological status, chronic diseases, and diet. Diet is one of the main causes of ID, and ID can occur when there is insufficient iron intake or dietary factors interfere with absorption (8). Therefore, a thorough understanding of children’s dietary habits is crucial for preventing ID. Dietary patterns refers to the types, quantities, and proportions of various foods in daily diet (9), which provides a more comprehensive perspective for dietary analysis, helping to determine the impact of diet on the short-term and long-term health of the population. An investigation in Brazil found a correlation between vegetarian dietary patterns and diminished serum ferritin levels (10), while a study conducted in South Africa on children aged 5–12 also showed a positive correlation between plant protein and carbohydrate-rich diets and ID (11). Guangzhou is famous for Cantonese cuisine, which is a typical representative of the Eastern healthy dietary model and the South China dietary model (12). Our previous research found that the fast-food pattern was a risk factor for anemia in children and adolescents, while the meat and egg pattern was a protective factor (13), but the relationship between dietary patterns and iron reserve level in the body is still unclear. This study aims to explore the association between dietary patterns and ID among rural school-aged children and adolescents in Guangzhou, China, in order to support the MDGs 2.2 to eliminate malnutrition.

## 2 Materials and methods

### 2.1 Participants

This cross-sectional study was conducted from June 2022 to May 2023. A multi-stage stratified cluster random sampling method was used to select research participants (1). Five primary schools, five middle schools and two high schools were randomly selected in rural areas of Guangzhou (2). Three grades were selected from each primary school, three grades were selected from each middle school, and one grade was selected from each high school (3). Two to four classes of students were randomly selected from each grade for primary schools, two to five classes of students were randomly selected from each grade for middle schools, and five to six classes of students were randomly selected from that grade for high schools.

**Figure 1:**
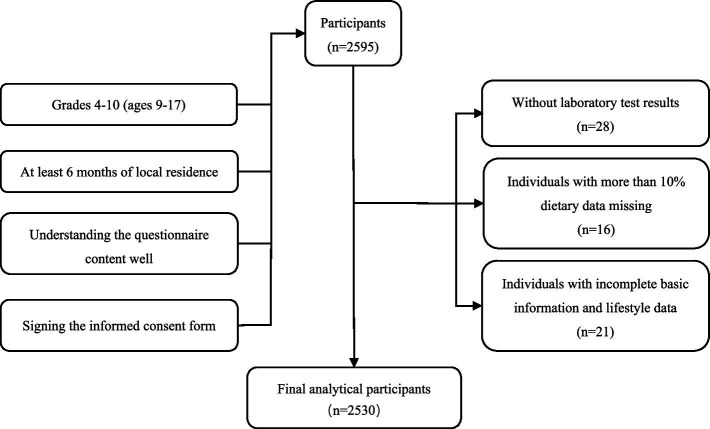
Flowchart of the selection of research participants.

The sample size was calculated using the formula: N= $deff\frac{\mu_{\alpha /2}^{2}P\left(1-P\right)}{\delta^{2}}\;$ (14). The meanings and values of each parameter are as follows: the confidence level is taken as 95%, and $\mu_{\alpha /2}=1.96$ . Because the definition criteria of ID in China are not clear and unified, the limits of each study for determining ID are different. Through the comprehensive judgment of multiple research literatures (2, 6, 15–17) and taking the average of multiple results, the ID detection rate of children and adolescents aged 6–17 years old in China is about 15%; the value of the design efficiency deff is taken as 2; the relative error *r* = 20%, *δ* = 20% × 15%. The sample size was 1,088 students according to the above formula. Considering the invalid questionnaires and rejection rate, the actual sample size was expanded by 10%, so at least 1,197 students were required for the survey. The screening process of the research participants is shown in Figure 1.

This study conducted a survey of 2,596 students. We excluded 28 students without the laboratory test results, 16 students with more than 10% missing dietary data (In the selected food items, data on consumption frequency or intake amounts were missing for more than 10%), and 21 students with incomplete basic information and lifestyle data. Ultimately, data from 2,530 students were included in the final analysis.

### 2.2 Survey content

Children and adolescents were interviewed face to face by the uniformly trained investigators. The survey content included questionnaires, physical examinations, and laboratory tests.

(1) Questionnaires: **①** Demographic information, including age, gender, education level of parents. **②** Lifestyle factors: boarding, smoking, alcohol consumption, moderate-intensity exercise, sleep duration and breakfast habits. **③** Dietary survey: a semi-quantitative food frequency questionnaire (FFQ) was used to investigate the frequency of food consumption and intake food consumed by children and adolescents in the past month. Food models and atlas were used to help participants assess their food intake. This FFQ was based on the food frequency questionnaire derived from the China National Chronic Non-Communicable Disease and Nutrition Surveillance in 2015 (18), which was partially adapted to the dietary characteristics of children and adolescents in Guangzhou by a panel of experts in the fields of epidemiology and nutrition. There was study has shown that the FFQ has fair reliability and validity (19), and it also has good consistency in a dietary survey of Guangzhou (20). According to the Chinese Food Composition Table Standard Edition (6th edition) (21), 66 types of food across 16 categories were included in this FFQ.
(2) Physical examinations: height and weight were measured by a mechanical height meter and an electronic scale, with measurements accurate to 0.1 cm and 0.1 kg, respectively. The testing instruments and procedures adhered to the Chinese national standard of anthropometric measurement methods in health surveillance (22). The nutritional status of children and adolescents was indicated by the Body Mass Index (BMI), calculated as BMI = weight (kg)/ height (m^2^) (23).
(3) Laboratory tests: serum ferritin (SF) was measured by the Latex-enhanced Immunoturbidimetric Assay. Serum transferrin (TRF) and C-reactive protein (CRP) was measured by the Immunoturbidimetric Assay.

### 2.3 Iron deficiency definition

Serum ferritin deficiency is assessed according to the Expert Consensus on Nutritional Prevention and Treatment of Iron Deficiency Anemia (24), in conjunction with CRP levels. For participants aged 5 years and older, CRP ≤ 5 mg/L and SF < 25 μg/L or CRP > 5 mg/L and SF < 32 μg/L is defined as SF deficiency, which was defined as iron deficiency.

Serum transferrin was used as an auxiliary indicator. According to the National Health Industry Standards of the People’s Republic of China (25), TRF > 3.6 g/L was defined as TRF over-standard, suggesting the reduction of iron in body.

### 2.4 Dietary pattern establishment

Dietary patterns were constructed through the exploratory factor analysis, 66 food items were classified into 16 food categories, as shown in Supplementary Table S1. The correlation matrix between the 16 food categories was statistically tested, with the Kaiser–Meyer–Olkin (KMO) test yielding >0.8 and the Bartlett’s sphericity test yielding a significant *p* (*p* < 0.001), indicating that the correlation between variables was sufficient for factor analysis. Principal component analysis was used to determine the common factors, and factor rotation was performed to minimize the correlation between factors. Factors used to describe different dietary patterns were identified based on the eigenvalue (>1), the scree plot, their professional significance and interpretability. Factors with absolute factor loadings >0.3 (26) were retained as components of dietary patterns (27) in this study. Composite factor scores were grouped into quartiles, with Q1, Q2, Q3, and Q4 representing scores from lowest to highest, respectively.

### 2.5 Statistical analysis

The questionnaires were coded uniformly. Epidata version 3.1 was used for double data input to establish the original database. Factor analysis was used to construct dietary patterns. Data were described as mean (95% CI) for continuous variables and described as n (%) for categorical variables. Continuous variables were tested for normality and compared by Mann–Whitney U test. Categorical variables were compared by chi-square test and chi-square trend test.

Due to the prevalence of ID was greater than 10% in this cross-sectional study, the relationship between ID and independent variables may be overestimated if the odds ratio (OR) is continued to be used to report the parsed results. In this case, prevalence ratio (PR) is the best indicator of association. Therefore, robust Poisson regression analysis was fitted to identify predictors of ID. PR and 95% CI were calculated, while the linear trend of PRs was estimated. A univariate analytical model and a multivariate analytical model were developed for each dietary pattern. The univariate analytical model was unadjusted. Based on the survey and references, the multifactor analytical model was adjusted for age, gender, BMI, smoking (28), moderate to high intensity exercise (29). And other factors that need to be adjusted were determined based on the results of the single-factor analysis.

**Figure 2:**
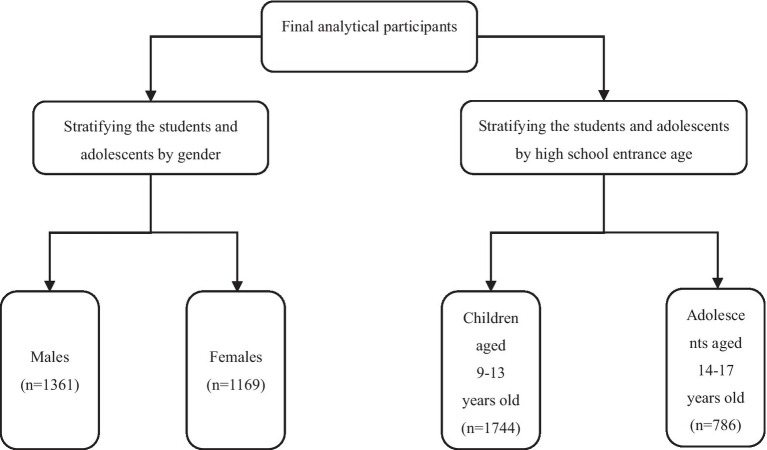
Flowchart of the establishment of stratified analysis subgroups.

Based on the existing studies, the prevalence of ID in girls was higher than in boys (30, 31), gender was selected as a stratification variable. Additionally, recognizing the nutrient needs of children at different stages (31) and the differences in mental development. Age was used as another stratification variable and high school entrance age (14 years old) (32) as the grouping criterion. Because SF was the main indicator, stratified analysis was conducted only on it. The detailed stratification process is shown in Figure 2. SPSS version 26.0 was used for all computations, and a *p*-value (two-sided) < 0.05 was considered to be statistically significant. GraphPad Prism 9.5 and Office 2021 were used for graphs.

## 3 Results

### 3.1 Participant characteristics

A total of 2,530 children and adolescents with complete data (53.79% male, *n* = 1,361; 46.21% female, *n* = 1,169) were included in this study. The age range of the children and adolescents was between 9 and 17 years with a mean age of 12.94 years. The prevalence of SF deficiency was 13.36%, with 5.80% in boys and 22.16% in girls. Compared with non-SF-deficient counterparts, the children and adolescents with SF deficiency were more likely to be female, older, and have shorter sleep duration (*p* < 0.05). The ratio of serum transferrin over-standard was 32.06%, as shown in Table 1. Compared with the normal group, the children and adolescents with TRF over-standard were more likely to be female, higher BMI, have less breakfast and the high school of education level of mother (*p* < 0.05).

**Table 1:** Demographic information and lifestyle characteristics of the children and adolescents aged 9–17 years in rural Guangzhou between June 2022 and May 2023.

Variable	Total	Serum ferritin deficiency		*P*	Transferrin over-standard		*P*
		Yes	No		Yes	No	
**Age in years, mean (95% CI)**	12.94 (12.86, 13.02)	13.46 (13.30,13.63)	12.86 (12.78,12.95)	**<0.001**	12.92 (12.79, 13.05)	12.95 (12.85, 13.05)	0.713
**Sex, *n* (%)**							
Male	1,361	79 (5.80)	1,282 (94.20)	**<0.001**	396 (29.10)	965 (70.90)	**0.001**
Female	1,169	259 (22.16)	910 (77.84)		415 (35.50)	754 (64.50)	
**BMI, mean (95% CI)**	18.91 (18.76, 19.05)	18.72 (18.43, 19.00)	18.93 (18.78, 19.09)	0.194	19.41 (19.15, 19.68)	18.67 (18.50, 18.83)	**<0.001**
**Boarding, *n* (%)**							
Yes	1,239	169 (13.64)	1,070 (86.36)	0.685	400 (32.28)	839 (67.72)	0.809
No	1,291	169 (13.09)	1,122 (86.91)		411 (31.84)	880 (68.16)	
**Education level of father, *n* (%)**							
Primary or below	86	11 (12.79)	75 (87.21)	0.173	20 (23.26)	66 (76.74)	0.238
Middle school	1,034	156 (15.09)	878 (84.91)		331 (32.01)	703 (67.99)	
High school	732	96 (13.11)	636 (86.89)		226 (30.87)	506 (69.13)	
College or above	612	70 (11.44)	542 (88.56)		210 (34.31)	402 (65.69)	
Unknown	66	5 (7.58)	61 (92.42)		24 (36.36)	42 (63.64)	
**Education level of mother, *n* (%)**							
Primary or below	133	20 (15.04)	113 (84.96)	0.14	32 (24.06)	101 (75.94)	**0.024**
Middle school	1,105	164 (14.84)	941 (85.16)		337 (30.50)	768 (69.50)	
High school	613	82 (13.38)	531 (86.62)		221 (36.05)	392 (63.95)	
College or above	620	67 (10.81)	553 (89.19)		206 (33.23)	414 (66.77)	
Unknown	59	5 (8.47)	54 (91.53)		15 (25.42)	44 (74.58)	
**Tried smoking, *n* (%)**							
Yes	146	13 (8.90)	133 (91.10)	0.114	40 (27.40)	106 (72.60)	0.214
No	2,384	325 (13.63)	2059 (86.37)		771 (32.34)	1,613 (67.66)	
**Alcohol consumption, *n* (%)**							
Yes	367	57 (15.53)	310 (84.47)	0.182	120 (32.70)	247 (67.30)	0.776
No	2,163	281 (12.99)	1882 (87.01)		691 (31.95)	1,472 (68.05)	
**Moderate-to-high-intensity exercise, *n* (%)**							
Less 3 times a week	1,146	150 (13.09)	996 (86.91)	0.716	389 (33.94)	757 (66.06)	0.064
3 times a week and more	1,384	188 (13.58)	1,196 (86.42)		422 (30.49)	962 (69.51)	
**Sleep duration, mean (95% CI)**	9.13 (9.08, 9.19)	8.76 (8.62, 8.89)	9.19 (9.14, 9.25)	**<0.001**	9.06 (8.97, 9.15)	9.17 (9.10, 9.23)	0.067
**Breakfast habits, *n* (%)**							
0–3 times a week	194	22 (11.34)	172 (88.66)	0.582	86 (49.33)	108 (55.67)	**<0.001**
4–6 times a week	387	56 (14.47)	331 (85.53)		133 (34.37)	254 (65.63)	
1 time a day and more	1949	260 (13.34)	1,689 (86.66)		592 (30.37)	1,357 (69.63)	
**Total**	2,530	338 (13.36)	2,192 (86.64)		811 (32.06)	1719 (67.94)	

Data are shown as number of cases (%) for categorical variables and mean (95% CI) for continuous variables. Mann–Whitney U test and Chi-square trend test were used for analysis. The bold *p*-value means “<0.05”.

### 3.2 Dietary patterns

Dietary patterns, determined by principal component analysis, as depicted in Table 2 and Supplementary Figure S1. Four dietary patterns were generated and explained by factor loading and component analysis. Factor analysis selected four major dietary patterns from the 16 food groups, accounting for 13.06% (snack and fast-food pattern), 12.41% (fruit and vegetable pattern), 10.88% (cereal and tuber pattern) and 9.32% (meat and offal pattern) of variance, which together accounted for 45.66% of the total variance. The factor loading matrix of the food groups was obtained by the varimax rotation, as shown in Table 3. Snack and fast-food pattern mainly includes snack food, fast food, beverages and candy. Fruit and vegetable pattern mainly includes fresh fruits, fresh vegetables, mushrooms, algae, and nuts. Cereal and tuber pattern mainly includes grains, potatoes, beans, bean products and eggs. Meat and offal pattern mainly includes poultry, offal and red meat.

**Table 2:** Factor loadings and dietary patterns for 16 food groups obtained by factor analysis.

Food group	Snack and fast-food pattern	Fruit and vegetable pattern	Cereal and tuber pattern	Meat and offal pattern
Snack food	0.669			
Fast food	0.662			
Beverages	0.650		0.317	
Candy	0.623			
Fresh fruits		0.747		
Fresh vegetables		0.699		
Mushrooms and algae		0.527	0.325	
Nuts	0.357	0.454		
Aquatic products		0.410		
Grain and potatoes			0.677	
Beans and bean products			0.666	
Eggs			0.507	
Milk and dairy			0.369	
Poultry				0.705
Offal				0.675
Red meat				0.556

PCA (principal component analysis) was used for analysis. Factor loadings of < 0.3 in absolute terms were excluded for simplicity.

**Table 3:** Analysis of the correlation between dietary patterns and serum ferritin of the children and adolescents aged 9–17 years in rural Guangzhou between June 2022 and May 2023.

**Dietary pattern**	**SF Deficiency**		***P***	***r***	**Model 1**		**Model 2**	
	**Yes**	**No**			**PR (95%CI)**	***P***	**PR (95%CI)**	***P***
**Snack and fast-food pattern, *n* (%)**								
Q1	68 (10.7)	565 (89.3)	**0.028**	0.044	1		1	
Q2	90 (14.2)	542 (85.8)			1.326 (0.987,1.780)	0.061	1.252 (0.946,1.659)	0.116
Q3	81 (12.8)	552 (87.2)			1.191 (0.880,1.613)	0.258	1.107 (0.825,1.485)	0.498
Q4	99 (15.7)	533 (84.3)			1.458 (1.093,1.946)	**0.010**	1.467 (1.110,1.937)	**0.007**
**Fruit and vegetable pattern, *n* (%)**								
Q1	93 (14.7)	540 (85.3)	**0.016**	−0.048	1		1	
Q2	94 (14.9)	538 (85.1)			1.012 (0.777, 1.319)	0.928	0.932 (0.726,1.198)	0.583
Q3	86 (13.6)	546 (86.4)			0.926 (0.706,1.215)	0.580	0.826 (0.636,1.074)	0.153
Q4	65 (10.3)	568 (89.7)			0.699 (0.519,0.941)	**0.018**	0.717 (0.538,0.956)	**0.023**
**Cereal and tuber pattern, *n* (%)**								
Q1	92 (14.5)	541 (85.5)	0.107	−0.032	1		1	
Q2	96 (15.2)	536 (84.8)			1.045 (0.803,1.361)	0.743	1.162 (0.904,1.494)	0.242
Q3	70 (11.1)	563 (88.9)			0.761 (0.569,1.018)	0.065	0.945 (0.713,1.252)	0.693
Q4	80 (12.7)	552 (87.3)			0.871 (0.659,1.151)	0.331	1.352 (1.027,1.779)	**0.032**
**Meat and offal pattern, *n* (%)**								
Q1	110 (17.4)	523 (82.6)	**<0.001**	−0.093	1		1	
Q2	97 (15.3)	535 (84.7)			0.883 (0.688,1.134)	0.330	0.888 (0.698,1.130)	0.335
Q3	73 (11.6)	559 (88.4)			0.665 (0.505,0.875)	**0.004**	0.682 (0.522,0.891)	**0.005**
Q4	58 (9.2)	575 (90.8)			0.527 (0.391,0.711)	**<0.001**	0.617 (0.458,0.831)	**0.001**

Chi-square trend test and robust Poisson regression analyses were used. Q1 is the reference group, model 1: unadjusted for covariates; model 2: adjusted for age, gender, BMI, moderate to high intensity exercise, sleep duration and smoking. The *r* is the Pearson correlation coefficient. The bold *p*-value means “<0.05”.

### 3.3 Characteristics of quartiles (Q) of dietary patterns in study participants

The characteristics of the Q1 (the lowest propensity) and Q4 (the highest propensity) quartiles of the four dietary patterns are shown in Supplementary Table S2. The analyses found that children and adolescents in Q4 group of snack and fast-food pattern were more likely to be older, males, have lower BMI, be boarding, have low parental education level, have the experience with smoking and alcohol, do more moderate to high intensity exercise, have shorter sleep duration and have less times of breakfast. Children and adolescents in Q4 group of fruit and vegetable pattern were more inclined to be younger, be boarding, have high parental education level, less drink and smoking, and have longer sleep duration. Children and adolescents in Q4 group of cereal and tuber pattern were more likely to be older, males, have a higher BMI, do more moderate to high intensity exercise and have more times of breakfast. Children and adolescents in Q4 group of meat and offal pattern were more likely to be older, males, have a higher BMI, be boarding, have high parental education level, have shorter sleep duration, and drink alcohol.

### 3.4 Association analysis between dietary patterns and iron deficiency

#### 3.4.1 Analysis of dietary patterns and serum ferritin

As shown in Table 3, the results of the Mantel–Haenszel chi-square test showed that the level of tendency for snack and fast-food pattern, fruit and vegetable pattern, and meat and offal pattern were all found to have a linear relationship with the risk of SF (*p* < 0.05). The results of the Pearson correlation showed that in snack and fast-food pattern *r* = 0.044, with a *p* < 0.05, which suggested that the higher the tendency toward this pattern, the more prevalent of SF deficient. Additionally, the results of the Pearson correlation showed that in the fruit and vegetable pattern *r* = −0.048, and in the meat and offal pattern *r* = −0.093, both with *p* < 0.05. These suggested that the higher the tendency toward fruit and vegetable pattern and meat and offal pattern, respectively, the less severe the lack of SF.

Robust Poisson regression analysis showed that after adjusting for age, gender, BMI, moderate to high intensity exercise, sleep duration and smoking, children and adolescents in the Q4 group (the highest propensity) of snack and fast-food pattern had a higher risk of SF deficiency than the Q1 group (the low propensity) (*PR* = 1.467, 95%CI: 1.110 ~ 1.937, *p* = 0.007). Children and adolescents in the Q4 groups of cereal and tuber pattern had a higher risk of SF deficiency than the Q1 group (*PR* = 1.352, 95%CI: 1.027 ~ 1.779, *p* = 0.032). Conversely, Children and adolescents in the Q4 groups of fruit and vegetable pattern had a lower risk of SF deficiency than the Q1 group (*PR* = 0.717, 95%CI: 0.538 ~ 0.956, *p* = 0.023). And both children and adolescents in the Q4 and Q3 groups of meat and offal pattern had a lower risk of SF deficiency than the Q1 group (*PR* = 0.617, 95%CI: 0.458 ~ 0.831, *p* = 0.001), (*PR* = 0.682, 95%CI: 0.522 ~ 0.891, *p* = 0.005).

#### 3.4.2 Analysis of dietary patterns and serum transferrin

As shown in Table 4, the results of the Mantel–Haenszel chi-square test showed that the level of tendency for meat and offal pattern was found to have a linear relationship with the risk of TRF (*p* < 0.05). The results of the Pearson correlation showed that in meat and offal pattern *r* = −0.051, with a *p* < 0.05, which suggested that the higher the tendency toward this pattern, the lower the degree to the TRF over-standard.

**Table 4:** Analysis of the correlation between dietary patterns and serum transferrin of the children and adolescents aged 9–17 years in rural Guangzhou between June 2022 and May 2023.

**Dietary pattern**	**Transferrin Over-standard**		***P***	***r***	**Model 1**		**Model 2**	
	**Yes**	**No**			**PR (95%CI)**	***P***	**PR (95%CI)**	***P***
**Snack and fast-food pattern, *n* (%)**								
Q1	194 (30.6)	439 (69.4)	0.450	0.015	1		1	
Q2	209 (33.1)	423 (66.9)			1.079 (0.918,1.268)	0.356	1.098 (0.935,1.289)	0.254
Q3	197 (31.1)	436 (68.9)			1.015 (0.861,1.197)	0.855	1.052 (0.894,1.238)	0.543
Q4	211 (33.4)	421 (66.6)			1.089 (0.928,1.279)	0.297	1.142 (0.972,1.341)	0.106
**Fruit and vegetable pattern, *n* (%)**								
Q1	211 (33.3)	422 (66.7)	0.607	−0.010	1		1	
Q2	199 (31.5)	433 (68.5)			0.945 (0.806, 1.108)	0.483	0.949 (0.810,1.111)	0.514
Q3	199 (31.5)	433 (68.5)			0.945 (0.806,1.108)	0.483	0.901 (0.769,1.056)	0.197
Q4	202 (31.9)	431 (68.1)			0.957 (0.817,1.122)	0.590	0.920 (0.786,1.078)	0.302
**Cereal and tuber pattern, *n* (%)**								
Q1	201 (31.8)	432 (68.2)	0.914	0.002	1		1	
Q2	209 (33.1)	423 (66.9)			1.041 (0.888,1.221)	0.617	1.096 (0.935,1.286)	0.258
Q3	193 (30.5)	440 (69.5)			0.960 (0.815,1.131)	0.627	1.030 (0.874,1.214)	0.721
Q4	208 (32.9)	424 (67.1)			1.036 (0.884,1.216)	0.660	1.147 (0.971,1.354)	0.107
**Meat and offal pattern, *n* (%)**								
Q1	222 (35.1)	411 (64.9)	**0.010**	−0.051	1		1	
Q2	212 (33.5)	420 (66.5)			0.956 (0.821,1.114)	0.567	0.986 (0.848,1.146)	0.850
Q3	194 (30.7)	438 (69.3)			0.875 (0.747,1.025)	0.098	0.905 (0.773,1.059)	0.213
Q4	183 (28.9)	450 (71.1)			0.824 (0.701,0.969)	**0.019**	0.841 (0.714,0.992)	**0.040**

Chi-square trend test and robust Poisson regression analyses were used. Q1 is the reference group, model 1: unadjusted for covariates; model 2: adjusted for age, gender, BMI, moderate to high intensity exercise, smoking, education level of mother and breakfast habits. The *r* is the Pearson correlation coefficient. The bold *p*-value means “<0.05”.

Robust Poisson regression analysis showed that after adjusting for age, gender, BMI, moderate to high intensity exercise, smoking, education level of mother and breakfast habits, children and adolescents in the Q4 groups of meat and offal pattern had a lower risk of TRF over-standard than the Q1 group (*PR* = 0.841, 95%CI: 0.714 ~ 0.992, *p* = 0.040).

#### 3.4.3 Analysis of dietary patterns and serum ferritin of different genders and ages

**Figure 3:**
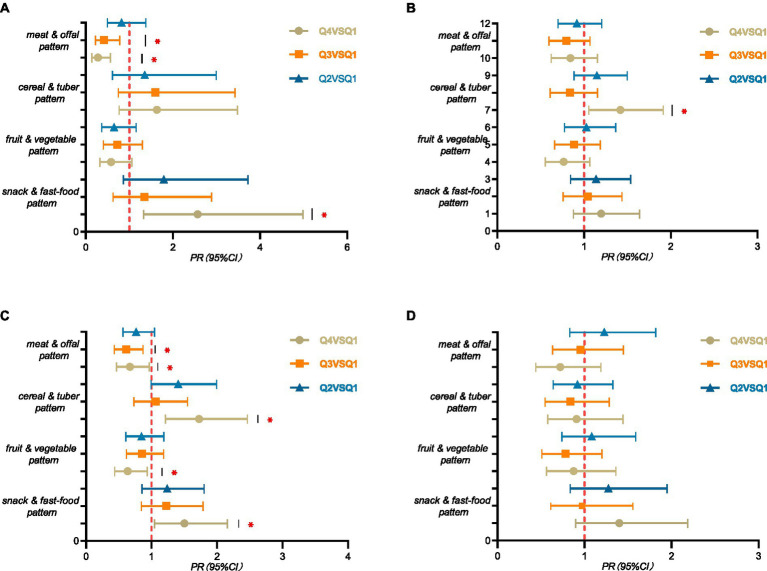
Layered analysis of serum ferritin among children and adolescents aged 9–17 years in rural Guangzhou between June 2022 and May 2023. PR (95% CI) for the highest propensity group (Q4), the third group (Q3), and the second group (Q2) compared to the lowest propensity group (Q1) for the four dietary patterns using robust Poisson regression analysis. **(A)** males, *n* = 1,361; **(B)** females, *n* = 1,169; **(C)** 9–13 years of age, *n* = 1744; **(D)** 14–17 years of age, *n* = 786. All models were adjusted for variables such as age or sex and BMI, moderate to high intensity exercise, sleep duration and smoking. “*” means *p*-value <0.05. The detailed tables are in Supplementary Table S3.

The results of stratified analysis were shown in Figure 3. After stratifying by gender, boys in Q4 group of snack and fast-food pattern had a higher risk of SF deficiency than the Q1 group (*PR* = 2.569, 95%CI: 1.324 ~ 4.984, *p* = 0.005). Whereas, boys in Q4 and Q3 groups of meat and offal pattern had a lower risk of SF deficiency than the Q1 group (*PR* = 0.279, 95%CI: 0.138 ~ 0.565, *p* < 0.001), (*PR* = 0.414, 95%CI: 0.222 ~ 0.774, *p* = 0.006) (Figure 3A). Meanwhile, girls in Q4 group of cereal and tuber pattern were more likely to SF deficiency (*PR* = 1.420, 95%CI: 1.054 ~ 1.912, *p* = 0.021) than the Q1 group (Figure 3B).

After stratifying by age, children aged 9–13 years in Q4 group of snack and fast-food pattern were more likely to SF deficiency than the Q1 group (*PR* = 1.501, 95%CI: 1.046 ~ 2.154, *p* = 0.028). Children aged 9–13 years in Q4 group of cereal and tuber pattern were more likely to SF deficiency than the Q1 group (*PR* = 1.725, 95%CI: 1.210 ~ 2.459, *p* = 0.003). Whereas children aged 9–13 years in Q4 group of fruit and vegetable pattern were less likely to SF deficiency than the Q1 group (*PR* = 0.637, 95%CI: 0.436 ~ 0.933, *p* = 0.021). Children aged 9–13 years in Q4 and Q3 group of meat and offal pattern were less likely to SF deficiency than the Q1 group (*PR* = 0.669, 95%CI: 0.464 ~ 0.963, *p* = 0.031), (*PR* = 0.611, 95%CI: 0.429 ~ 0.869, *p* = 0.006) (Figure 3C).

## 4 Discussion

Iron deficiency and iron deficiency anemia have caused a huge disease burden worldwide, threatening the lives and health of billions of people, and are also one of the five major causes of disease burden worldwide (2). In this study, the overall prevalence of ID among the participants was 13.36%, with rates of 5.8% in boys and 22.16% in girls. And the rate of transferrin over-standard was 32.06%. This prevalence of ID was similar to that of 12.60% among children aged 12–17 reported in Beijing (17). In addition, it is higher than the prevalence of 8.19% reported in a survey of children aged 6–12 years in Guangzhou (16), but lower than the prevalence of 18.64% in children aged 8–12 years in Lanzhou, China (15). And it also lower than the prevalence of 20.9% in adolescents aged 10–19 years in India (30). This difference may be caused by the different economic levels and demographic differences in population in different regions. However, the ratio of TRF over-standard was higher than prevalence of ID, possibly because in diagnosing ID, TRF has a higher sensitivity and a lower specificity than SF (2).

This study observed a higher prevalence of ID among girls compared to boys. This phenomenon may be attributed to the gradual maturation of sexual development in girls, leading to increased iron loss during menstruation (33). Furthermore, the prevalence of ID was higher among the older children and adolescents. This could be attributed to the accelerated growth and development during puberty (34), which leads to increased nutritional requirements and increases the possibility of nutritional deficiency. The results were in line with the findings from a previous study (31). The study also found that longer sleep duration was negatively correlated with ID, which was consistent with findings from a study in the United States linking ID to poor sleep quality (35).

Moreover, higher BMI and less breakfast were all associated with the higher rate of transferrin over-standard. Obesity was associated with higher levels of inflammation, which would affect the iron levels in body (36). And skipping breakfast might cause inadequate nutritional intake, which would lead to ID (37). The study also indicated a positive correlation between maternal education level and TRF over-standard. This was attributed to the fact that mothers with higher education levels typically prioritize career advancement, leading to an inadequate balance between work and family. Consequently, they had relatively less time to prepare home-cooked meals or tend to rely more on convenience foods (38). Prolonged consumption of these low-iron foods by children increases the likelihood of ID in body.

In this study, four dietary patterns were identified, including snack and fast-food pattern, fruit and vegetable pattern, cereal and tuber pattern, and meat and offal pattern. Notably, the cumulative contribution rate of snack and fast-food pattern was the highest (13.06%), which indicates that the eating habits of children and adolescents at this stage are significantly influenced by Western dietary culture (39). On the contrary, the other three dietary patterns reflected many characteristics of Cantonese cuisine. Cantonese cuisine forms a significant component of the Lingnan dietary pattern (40). Thus, this study holds crucial practical significance for providing nutritional guidance to children and adolescents with iron deficiency or insufficiency in Guangzhou and even the Lingnan region.

In this study, we found that both snack and fast-food pattern and cereal and tuber pattern were positively associated with the risk of ID. Snack and fast-food pattern was characterized by a higher intake of snack food, fast food, and beverages; cereal and tuber pattern was characterized by a higher intake of grains, potatoes and beans. The common feature of these two dietary patterns is that the food they contain either has low iron content or may interfere with iron absorption. For example, all foods of snack and fast-food pattern were generally of lower nutritional value, mainly in the form of higher energy density and saturated fat content, as well as higher added sugars and salt. Prolonged consumption of these foods could impair the ability of liver cells to absorb iron (41). And the sugary beverages could negatively affect iron metabolism, thereby reducing ferritin levels in the body (42). Moreover, these types of foods are actually low in iron. A study in Poland (43) showed that fast foods provided only 4% of the body’s recommended daily iron intake. At the same time, fast foods on the market were mainly composed of white meat such as fried chicken breast and chicken nuggets, which could not provide enough iron (12). In addition, the effect of cereal and tuber pattern was also consistent with the results in the existing studies, such as diet based on rice and wheat or whole wheat bread led to the development of ID (44–46). This might be cause by the fact that most of the food in this dietary pattern was the staple food. However, these plant foods contained anti-nutritional factors such as phytic acid and tannin, which could form insoluble complexes with iron, thus reducing the bioavailability and intestinal absorption of iron. Moreover, iron in plant foods was mainly non-heme iron, and its bioavailability was lower than that of heme iron in animal foods (47). Therefore, even if the intake was high, the actual amount of iron absorbed and utilized by the human body might be insufficient.

Conversely, we found that both fruit and vegetable pattern and meat and offal pattern were negatively associated with the risk of ID. Fruit and vegetable pattern, which was characterized by a higher intake of fresh fruits and vegetables; meat and offal pattern, which was characterized by a higher intake of offal, red meat, and poultry. Both of the two dietary patterns contained foods that were high in iron or contained ingredients that facilitated iron absorption. For example, fresh fruits and vegetables were naturally rich sources of vitamin C, which played an important role in the absorption of iron. It not only enhanced the absorption of non-heme iron (48), but also promoted the export of iron from the gut into the circulatory system by altering the expression of hepcidin RNA in cells (49). A study also had shown that girls who did not eat enough fruit were at higher risk of low serum iron levels, and that eating green leafy vegetables such as amaranth could significantly increase serum hepcidin levels in girls, which could help prevent ID (50). What’s more, the foods such as meat and offal are rich in iron (8, 51), predominantly in the form of heme iron. Heme iron was a kind of stable iron combined with porphyrin in hemoglobin and myoglobin, which could be directly absorbed by intestinal mucosal epithelial cells. Therefore, it was less affected by other dietary factors and showed high bioavailability. A study conducted in Britain (52) was also indicated that increased consumption of animal foods would reduce the risk of ID. Moreover, the transferrin over-standard was only negatively associated with meat and offal pattern, providing further evidence that this pattern was protective for ID.

However, the three Pearson correlations between dietary patterns and SF and the one Pearson correlation between dietary patterns and TRF were weak in this study. This could be explained by two main factors. Firstly, the mechanisms of iron storage and release in the body involved multiple physiological processes, which were not a simple linear process. Secondly, the composition of the daily diet was complex, with components that both affected and promoted iron absorption. These two factors might be in a dynamic equilibrium state, which made the association between diet and iron reserves weak.

The multivariate analysis on the association between dietary pattern and TRF indicated that only meat and offal pattern was correlated with TRF. Children and adolescents who were more inclined toward meat and offal pattern had a lower risk of TRF over-standard. This was consistent with the effect of dietary patterns on SF, both indicated that meat and offal pattern was associated with a lower incidence of ID. And it implied that meat and offal pattern had the most pronounced effect on iron levels in the body, which was also in line with previous research (8) stating that meat and offal were good sources of iron intake.

In addition, the results of the stratified study showed that the negative effect of snack and fast-food pattern and the protective benefits of meat and offal pattern are more obvious for boys. Boys were more likely to choose fast foods and meat as food sources because they were more physically active and had higher energy expenditure. These foods were usually high in calories and protein, which could help them recover quickly and maintained the energy level needed for daily activities. A study conducted in Canadia (53) also indicated that men consumed fast foods more frequently than women. And the negative effect of cereal and tuber pattern was more obvious for girls. It was speculated that some girls might choose a vegetarian diet to maintain a slim figure, however, long-term vegetarian diet might lead to inadequate iron intake, which in turn affected iron reserves in the body (54). The negative effect or protective benefits of the four dietary patterns were obvious for children aged 9–13. This phenomenon might be related to the weak self-control ability and low cognitive development level of children in this age group (55). They might be more susceptible to the temptation of instant gratification and chose to eat more of their preferred foods. In addition, the cognitive abilities of children aged 9–13 were still developing compared with older children (56), which might affect their understanding of the importance of healthy diet. This result also indicated that early nutrition and health education for children is better.

However, the present study had several limitations. Firstly, the dietary data were collected based on the dietary intake of children and adolescents over the past month, and recall bias cannot be avoided. Secondly, the study only utilized two indicators for ID, which may not provide a comprehensive assessment. Further research could be conducted by incorporating additional indicators. Thirdly, the participants of this study were children and adolescents from the rural areas of Guangzhou. Therefore, the results need to be extrapolated with caution due to the potential differences in population structure and the dietary habits characteristic of the local area. Finally, the cross-sectional design of the study could not confirm causation. Prospective cohort studies are needed to clarify the relationship between dietary patterns and ID.

## 5 Conclusion

To sum up, the situation of iron deficiency among rural school-age children and adolescents in Guangzhou remains a significant concern. Four dietary patterns were identified in this study, including snack and fast-food pattern, fruit and vegetable pattern, cereal and tuber pattern, and meat and offal pattern. Snack and fast-food pattern and cereal and tuber pattern are risk factors for ID, and fruit and vegetable pattern and meat and offal pattern are protective factors for ID. The impact of diet on body iron levels is more obvious in boys and younger children. The findings of this study can provide evidence for formulating prevention and control measures on adolescent iron deficiency and iron deficiency anemia.

## Data Availability

The original contributions presented in the study are included in the article/Supplementary material, further inquiries can be directed to the corresponding author.

## References

[1] Georgieff MK Krebs NF Cusick SE. The benefits and risks of Iron supplementation in pregnancy and childhood. Annu Rev Nutr. (2019) 39:121–46. doi: 10.1146/annurev-nutr-082018-124213, PMID: 31091416 PMC7173188

[2] Qin R He S Yin S Chai Y. Expert consensus on the prevention and treatment of iron deficiency and iron deficiency anemia in children. Chin J Woman Child Health Res. (2023) 34:1–11. doi: 10.3969/j.issn.1673-5293.2023.06.001

[3] World Health Organization. WHO guidance helps detect iron deficiency and protect brain development. (2020). Available at: https://www.who.int/news/item/20-04-2020-who-guidance-helps-detect-iron-deficiency-and-protect-brain-development (Accessed March 15, 2024).

[4] UN Secretary-General. Agriculture development, food security and nutrition report of the secretary-general United Nations. (2017). Available at: https://digitallibrary.un.org/record/3937115?v=pdf (Accessed March 15, 2024).

[5] Liu J Liu G Li Y Wen R Wang D. Meta-analysis on prevalence of iron deficiency anemia in Chinese children aged 0-14 years from 2000 to 2020. Chin J School Health. (2020) 41:1876–81. doi: 10.16835/j.cnki.1000-9817.2020.12.028

[6] Ma D Zhang Y You L Tuo Y Sheng Q Wang P. Analysis on the iron deficiency and the rate of anemia of 3-11 years old children in 7 cities and 2 countryside in China. J Hygiene Res. (2014) 43:224–7. doi: 10.19813/j.cnki.weishengyanjiu.2014.02.011 24868973

[7] Red Blood Cell Disease (Anemia)Group. Multidisciplinary expert consensus on the diagnosis, treatment and prevention of iron deficiency and iron deficiency anemia (edition 2022). Nat Med J China. (2022) 102:3246–56. doi: 10.3760/cma.j.cn112137-20220621-01361

[8] Zimmermann M Hurrell R. Nutritional iron deficiency. Lancet. (2020). 370:511–20. doi: 10.1016/S0140-6736(07)61235-5 17693180

[9] Teixeira B Afonso C Rodrigues S Oliveira A. Healthy and sustainable dietary patterns in children and adolescents: a systematic review. Adv Nutr. (2022) 13:1144–85. doi: 10.1093/advances/nmab148, PMID: 34850824 PMC9340991

[10] Slywitch E Savalli C Duarte A Escrivão M. Iron deficiency in vegetarian and omnivorous individuals: analysis of 1340 individuals. Nutrients. (2021) 13:2964. doi: 10.3390/nu13092964 34578841 PMC8468774

[11] Visser M van Zyl T Hanekom SM Baumgartner J van der Hoeven M Taljaard-Krugell C. Nutrient patterns and their relation to anemia and iron status in 5- to 12-y-old children in South Africa. Nutrition. (2019) 62:194–200. doi: 10.1016/j.nut.2019.01.016, PMID: 30925444

[12] Giromini C Givens DI. Benefits and risks associated with meat consumption during Key life processes and in relation to the risk of chronic diseases. Food Secur. (2022) 11:2063. 10.3390/foods11142063 PMC9318327 35885304

[13] Ma J Huang J Zeng C Zhong X Zhang W Zhang B. Dietary patterns and association with Anemia in children aged 9–16 years in Guangzhou, China: A Cross-Sectional Study. Nutrients. (2023). 15:4133. doi: 10.3390/nu15194133 PMC10574347 37836416

[14] Zhan S Ye D Tan H. Epidemiology. Beijing: People’s Medical Publishing House. (2017).

[15] Mu J. Analysis the prevalence rate of Iron deficiency and relevant factors of Iron deficiency Anemia aged 6~12 years old in Lanzhou and Dongxiang Lanzhou. [dissertation]. China (Gansu Province): Lanzhou University (2018).

[16] Huang H Luo H Li H Cheng Z Cheng M. Analysis on iron status among children aged 2 ~12 years in Guangzhou. Studies Trace Elements Health. (2023) 40:45–7.

[17] Huang L Yu B Sha Y Yao Y Tu R Zhao Y. Iron store status of children aged 6 to 17 years in Beijing in 2016-2017. Journal of Hygiene Research. (2023) 52:924–9. doi: 10.19813/j.cnki.weishengyanjiu.2023.06.011 38115656

[18] National Health Commission of the People’s Republic of China. (2014). Work plan for chronic disease and nutrition surveillance of Chinese residents (Trial). Available at: http://www.nhc.gov.cn/jkj/s5878/201409/9b0f5f9e50a9457fb54f140c6208997b.shtml (Accessed May 16, 2024).

[19] Huang L Xie R Ni C Yang M Wang Z Ding G. A Meta-analysis of the reproducibility and validity of food frequency questionnaires among Chinese adults. ACTA Nutrimenta SINICA. (2022) 44:293–300. doi: 10.13325/j.cnki.acta.nutr.sin.2022.03.008

[20] Huang L Luo X Tan Y Sui Y. Study on reproducibility and validity of food frequency questionnaire in Guangzhou. Chin J Disease Control Prevent. (2013) 17:711–4.

[21] Yang YX. China food composition tables standard. Edition I. Sixth Edition. Beijing: Peking University Medical Press (2022).

[22] National Health and Family Planning Commission of the People’s Republic of China. Anthropometric measurements method in health surveillance. Beijing: Standards Press of China (2013).

[23] National Disease Control and Prevention Administration. (2018). Screening for overweight and obesity among school-age children and adolescents. Available at: https://www.ndcpa.gov.cn/jbkzzx/c100202/common/content/content_1666364400852602880.html (Accessed May 16, 2024).

[24] Chinese Nutrition Society. Scientific consensus on screening, prevention and treatment of Iron deficiency Anemia. Acta Nutrimenta Sinica. (2019) 41:417–26. doi: 10.13325/j.cnki.acta.nutr.sin.2019.05.001

[25] National Health Commission of the People’s Republic of China. (2018). Reference intervals for common clinical biochemistry tests-Part 9:Serum C-reactiveprotein,prealbumin,transferrin,B2-microglobulin. Available at: http://www.nhc.gov.cn/wjw/s9492/201812/3728fb3be3fa40ba9cd82b2f4dc597f9.shtml (Accessed March 16, 2024).

[26] Wang Y Tian T Pan D Zhang J Xie W Wang S. The relationship between dietary patterns and overweight and obesity among adult in Jiangsu Province of China: a structural equation model. BMC Public Health. (2021) 21:1225. doi: 10.1186/s12889-021-11341-3, PMID: 34172040 PMC8229268

[27] Wang Y Xie W Tian T Zhang J Zhu Q Pan D. The relationship between dietary patterns and high blood glucose among adults based on structural equation modelling. Nutrients. (2022) 14. doi: 10.3390/nu14194111 PMC9570980 36235763

[28] Vivek A Kaushik RM Kaushik R. Tobacco smoking-related risk for iron deficiency anemia: a case-control study. J Addict Dis. (2023) 41:128–36. doi: 10.1080/10550887.2022.2080627 35699272

[29] Hinton PS. Iron and the endurance athlete. Appl Physiol Nutr Metab. (2014) 39:1012–8. doi: 10.1139/apnm-2014-0147 25017111

[30] Kulkarni B Peter R Ghosh S Pullakhandam R Thomas T Reddy GB. Prevalence of Iron deficiency and its sociodemographic patterning in Indian children and adolescents: findings from the comprehensive National Nutrition Survey 2016-18. J Nutr. (2021) 151:2422–34. doi: 10.1093/jn/nxab145, PMID: 34049401

[31] Wang L Huo J Chen D Man Q Tang Y Zhang J. Iron status among children aged 6-17 years by serum ferritin - China, 2016-2017. China CDC Wkly. (2021) 3:221–5. doi: 10.46234/ccdcw2021.063, PMID: 34594854 PMC8393040

[32] Chinese Nutrition Society, Chinese Dietary Guidelines for School-age Children. Version 1, April 2022 ed. 2022. Beijing: People’s Medical Publishing House (2022).

[33] Percy L Mansour D Fraser I. Iron deficiency and iron deficiency anaemia in women. Best Pract Res Clin Obstet Gynaecol. (2017) 40:55–67. doi: 10.1016/j.bpobgyn.2016.09.007, PMID: 28029503

[34] Tao B. Child and adolescent health. Beijing: People's Medical Publishing House (2017).

[35] Hinai M Jansen E Song P Peterson K Baylin A. Iron deficiency and vitamin D deficiency are associated with sleep in females of reproductive age: an analysis of NHANES 2005-2018 data. J Nutr. (2024) 154:648–57. doi: 10.1016/j.tjnut.2023.11.030 38042351 PMC10997906

[36] Aguree S Owora A Hawkins M Reddy M. Iron deficiency and Iron deficiency Anemia in women with and without obesity: NHANES 2001-2006. Nutrients. (2023) 15:2272. doi: 10.3390/nu15102272 37242155 PMC10223101

[37] Jalambo MO Karim NA Naser IA Sharif R. Prevalence and risk factor analysis of iron deficiency and iron-deficiency anaemia among female adolescents in the Gaza strip. Palestine Public Health Nutr. (2018) 21:2793–802. doi: 10.1017/S1368980018001568, PMID: 29911513 PMC10261006

[38] Wu JC. Parental work characteristics and diet quality among pre-school children in dual-parent households: results from a population-based cohort in Taiwan. Public Health Nutr. (2018) 21:1147–55. doi: 10.1017/S1368980017003548, PMID: 29233206 PMC10260848

[39] Zhai FY Du SF Wang ZH Du WW Popkin BM. Dynamics of the Chinese diet and the role of urbanicity, 1991-2011. Obes Rev. (2014) 15:16–26. doi: 10.1111/obr.12124 24341755 PMC3868998

[40] Cantonese Dietary Pattern Expert Group (2023). An Introduction to the cantonese dietary pattern (2023). Acta Nutrimenta Sinica. 45:417–21. doi: 10.13325/j.cnki.acta.nutr.sin.2023.05.015

[41] Dongiovanni P Lanti C Gatti S Rametta R Recalcati S Maggioni M. High fat diet subverts hepatocellular iron uptake determining dysmetabolic iron overload. PLoS One. (2015) 10:e0116855. doi: 10.1371/journal.pone.0116855, PMID: 25647178 PMC4315491

[42] Harder N Hieronimus B Stanhope K Shibata N Lee V Nunez M. Effects of dietary glucose and fructose on copper, Iron, and zinc metabolism parameters in humans. Nutrients. (2020) 12:2581. doi: 10.3390/nu12092581 32854403 PMC7551875

[43] Grajeta H Prescha A Biernat J. Fe, ca and mg contents in selected fast-food products in Poland. Nahrung. (2002) 46:7–10. doi: 10.1002/1521-3803(20020101)46:1<7::AID-FOOD7>3.0.CO;2-I PMID: 11890058

[44] Kehoe L Buffini M McNulty B Kearney J Flynn A Walton J. Food and nutrient intakes and compliance with recommendations in school-aged children in Ireland: findings from the National Children's food survey II (2017-2018) and changes since 2003-2004. Br J Nutr. (2023) 129:2011–24. doi: 10.1017/S0007114522002781 36047066 PMC10167663

[45] Marrone G Guerriero C Palazzetti D Lido P Marolla A Daniele F. Vegan diet health benefits in metabolic syndrome. Nutrients. (2021) 13:817. doi: 10.3390/nu13030817 33801269 PMC7999488

[46] O'Keefe JH O'Keefe EL Lavie CJ Cordain L. Debunking the vegan myth: the case for a plant-forward omnivorous whole-foods diet. Prog Cardiovasc Dis. (2022) 74:2–8. doi: 10.1016/j.pcad.2022.08.001, PMID: 35944662

[47] Peddie M Ranasinghe C Scott T Heath A Horwath C Gibson R. Dietary intake nutritional status and lifestyle of adolescent vegetarian and nonvegetarian girls in New Zealand (the SuNDiAL project): protocol for a clustered, cross-sectional survey. JMIR Res Protoc. (2020) 9:e17310. doi: 10.2196/17310 32459178 PMC7287748

[48] Saunders AV Craig WJ Baines SK Posen JS. Iron and vegetarian diets. Med J Aust. (2013) 199:S11–6. doi: 10.5694/mja11.11494 PMID: 25369923

[49] Chiu PF Ko SY Chang CC. Vitamin C affects the expression of hepcidin and erythropoietin receptor in HepG2 cells. J Ren Nutr. (2012) 22:373–6. doi: 10.1053/j.jrn.2011.09.007, PMID: 22227182

[50] Ghatpande NS Apte PP Naik SS Kulkarni PP. Fruit and vegetable consumption and their association with the indicators of Iron and inflammation status among adolescent girls. J Am Coll Nutr. (2019) 38:218–26. doi: 10.1080/07315724.2018.1492470, PMID: 30130470

[51] Yang Y Ge K. Encyclopedia of nutritional sciences. Beijing: People’s Medical Publishing House. (2019).

[52] Papier K Fensom GK Knuppel A Appleby PN Tong TYN Schmidt JA. Meat consumption and risk of 25 common conditions: outcome-wide analyses in 475,000 men and women in the UK biobank study. BMC Med. (2021) 19:53. doi: 10.1186/s12916-021-01922-9, PMID: 33648505 PMC7923515

[53] Nardocci M Leclerc BS Louzada ML Monteiro CA Batal M Moubarac JC. Consumption of ultra-processed foods and obesity in Canada. Can J Public Health. (2019) 110:4–14. doi: 10.17269/s41997-018-0130-x, PMID: 30238324 PMC6964616

[54] Haider LM Schwingshackl L Hoffmann G Ekmekcioglu C. The effect of vegetarian diets on iron status in adults: a systematic review and meta-analysis. Crit Rev Food Sci Nutr. (2018) 58:1359–74. doi: 10.1080/10408398.2016.1259210, PMID: 27880062

[55] Gülseven Z Yu MVB Zarrett N Vandell DL Simpkins SD. Self-control and cooperation in childhood as antecedents of less moral disengagement in adolescence. Dev Psychopathol. (2023) 35:290–300. doi: 10.1017/S0954579421000584, PMID: 34308803

[56] Breit M Brunner M Preckel F. General intelligence and specific cognitive abilities in adolescence: tests of age differentiation, ability differentiation, and their interaction in two large samples. Dev Psychol. (2020) 56:364–84. doi: 10.1037/dev0000876, PMID: 31886691

