# Supplementary material for: Dietary patterns and association with Iron deficiency among children and adolescents aged 9–17 years in rural Guangzhou, China: a cross-sectional study
Source: Front Nutr. 2024 Sep 2;11:1443849. doi: 10.3389/fnut.2024.1443849 (PMC11403371; doi:10.3389/fnut.2024.1443849)
Supplement: Supplementary file 1 [file Table_1.docx]

**Supplementary table 1. Food groups used in the factor analysis**

| **Number** | **Food Group** | **Examples of Food Items** |
| --- | --- | --- |
| 1 | Grain and potatoes | Rice and rice products, wheat, maize, potatoes, sweet potatoes |
| 2 | Beans and bean products | Soya bean, soya milk, tofu, dried bean curd |
| 3 | Fresh vegetables | Lettuce, tomato, broccoli, cabbage, cucumber |
| 4 | Mushrooms and algae | Mushrooms, nori, seaweed |
| 5 | Fresh fruits | Apples, bananas, pears, oranges |
| 6 | Milk and dairy products | Cow's milk, goat's milk, yoghurt, cheese, sliced milk |
| 7 | Red meat | Pork, beef, lamb, bacon, ham |
| 8 | Poultry | Chicken, duck, goose |
| 9 | Offal | Heart, liver, kidney, large intestine |
| 10 | Aquatic products | Fish, shrimps, crabs, clams |
| 11 | Eggs | Eggs, duck eggs, quail eggs |
| 12 | Nuts | Peanuts, almonds, walnuts, hazelnuts |
| 13 | Candy | Sugar, jams, jellies, candies, chocolates |
| 14 | Snack food | Spicy gluten, chips, fried puffed snacks |
| 15 | Fast food | Instant noodles, self-heating rice, hamburgers |
| 16 | Beverages | milk beverages, sweet tea beverages,  carbonated beverage, sports beverages |
