# Supplementary figures and images for: Dietary patterns and association with Iron deficiency among children and adolescents aged 9–17 years in rural Guangzhou, China: a cross-sectional study
Source: Front Nutr. 2024 Sep 2;11:1443849. doi: 10.3389/fnut.2024.1443849 (PMC11403371; doi:10.3389/fnut.2024.1443849)

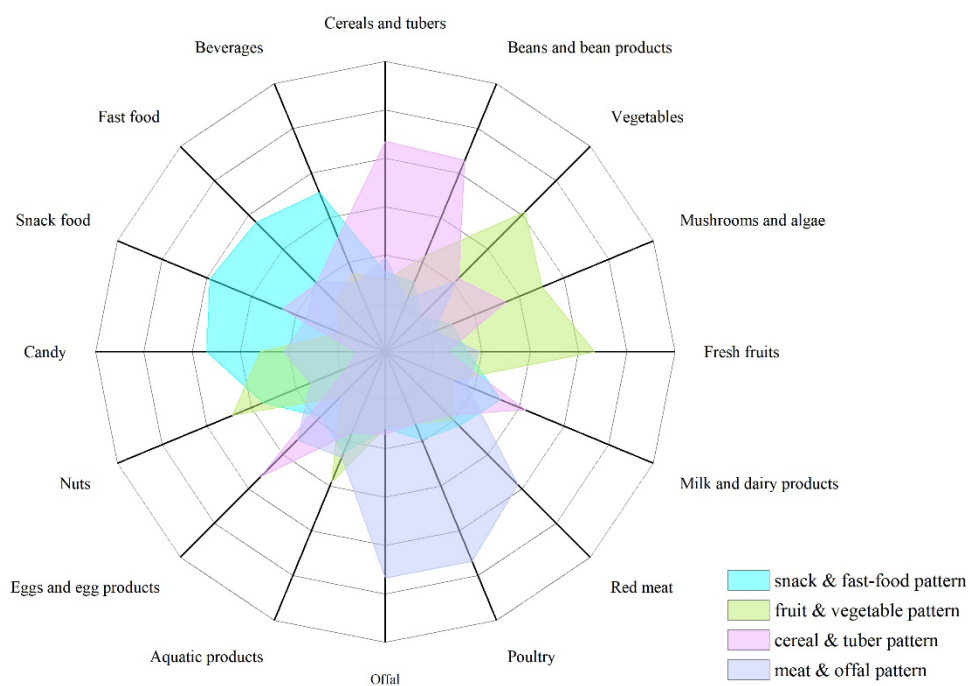

**Supplementary figure 1. Radar chart of different dietary patterns obtained by factor analysis**

Supplement: Supplementary file 4 [file Image_1.pdf]
